# Effectiveness of strenghtning oropharyngeal myofunctional therapy combined with cervical spine exercises in mild to moderate obstructive sleep apnoea

**DOI:** 10.1007/s11325-025-03487-w

**Published:** 2025-11-08

**Authors:** Teresa Díaz de Terán, Javier Bolado, Patricia Palomo, Pedro Muñoz, Carmen de Labra, Mónica González

**Affiliations:** 1 Department of Pneumology. Sleep and Non-Invasive Ventilation Unit, ’Marqués de Valdecilla’ University Hospital, University of Cantabria, ’Marqués de Valdecilla’ Institute for Research (IDIVAL), Santander, Spain; 2 Department of Nursing, University of Extremadura, Badajoz, Spain; 3 Rehabilitation Unit, ‘Marqués de Valdecilla’ University Hospital, Cantabrian Health Service, Santander, Spain; 4 Public Health Unit, Primary Care Management, Cantabrian Health Service, Santander, Spain; 5 Research, Health and Podiatry Group, Department of Physiotherapy, Medicine and Biomedical Sciences, Faculty of Nursing and Podiatry, Industrial Campus of Ferrol, Universidade da Coruña, Ferrol, Spain

**Keywords:** Oropharyngeal myofunctional therapy, Obstructive sleep apnoea, Apnoea-Hypopnoea index, Respiratory polygraphy

## Abstract

**Objective:**

To assess the effectiveness of an intervention programme combining Oropharyngeal Myofunctional Therapy (OMT) and cervical spine exercises in the general population with mild to moderate Obstructive Sleep Apnoea (OSA), analysing its impact on respiratory variables (AHI, MSatO_2_, ODI, TC90, Supine AHI), daytime sleepiness and quality of life.

**Materials and methods:**

A single-blind randomised clinical trial with 32 participants diagnosed with mild to moderate OSA, assigned into two groups: control (*n* = 16) and intervention (*n* = 16). Both groups received hygiene- and diet-related recommendations, and the intervention group completed an OMT programme and cervical spine exercises over 20 weeks (May 2023–November 2024). The respiratory variables were evaluated using respiratory polygraphy, and daytime sleepiness and quality of life were measured using the Epworth Sleepiness Scale and EuroQol-5D scales, respectively.

**Results:**

No statistically significant differences were found between groups (Median [IQR]): Apnoea–hypopnoea index (2.0 [-6/6], CI 95%, *p* = 0.86), Mean oxygen saturation (-0.5 [-1/0], CI 95%, *p* = 0.43), Oxygen Desaturation index (1.0 [-1/5], CI 95%, *p* = 0.72), Time with oxygen saturation below 90% (1.0 [0/3], CI 95%, *p* = 0.10), Epworth Sleepiness Scale score (-1.5 [-4/0], CI 95%, *p* = 0.83), and EuroQol-5D quality of life questionnaire (5.0 [0–10], CI 95% *p* = 0.08).

**Conclusion:**

The comprehensive 20-week OMT programme and cervical spine exercises showed no effectiveness in improving respiratory parameters, daytime sleepiness or quality of life in patients with mild to moderate OSA compared to hygiene- and diet-related measures alone. The null results observed in this study suggest relevant clinical implications, such as the limited efficacy of low-frequency OMT protocols.

**Supplementary Information:**

The online version contains supplementary material available at 10.1007/s11325-025-03487-w.

## Introduction

Obstructive Sleep Apnoea (OSA) is a common respiratory disorder characterised by the completed or narrowing and intermittent collapse of the upper airway (UA) during sleep, due to anatomical and functional alterations and associated with factors such as obesity, age and sex [1, 2]. This condition, which is estimated to affect up to 1 billion people worldwide and is usually 2 to 3 times more prevalent in men, has a significant prevalence in adults, causes intermittent hypoxia and changes in intrathoracic pressure, disrupts sleep (thus impairing its quality) and is associated with cardiovascular and cognitive risks, resulting in reduced quality of life [3–5]. The main etiological and pathophysiological factors contributing to the onset of OSA include anatomical narrowing of the upper airway, obesity, age, male sex, neuromuscular dysfunction, and diminished upper airway muscle tone. These factors may act singly or in combination, and their influence can vary between adults and specific populations such as children or individuals with comorbidities [6]. Classification based on age consists of pediatric population (0–18 years), young and middle-aged adults (18–65 years), and older adults (>65 years) [7].

Although standard treatment is continuous positive airway pressure (CPAP) [8], its poor compliance underlines the need for alternative therapies; it is estimated that approximately 50% of patients do not use CPAP as prescribed [9, 10].

Oropharyngeal Myofunctional Therapy (OMT) is a technique that has become a promising complementary therapy within a multi-disciplinary approach, aiming to strengthen the oropharyngeal muscles and reduce UA collapse [11–14]. Its use has been described in the treatment of various conditions, such as malocclusion, dysphagia, and temporomandibular joint disorders [15]. Recent systematic reviews and meta-analyses suggest that OMT may offer therapeutic benefits in patients with OSA, with minimal risk of complications. However, further studies are needed to evaluate long-term adherence and sustainability of effects [16, 17]. Various studies support its effectiveness, particularly in mild to moderate OSA, as a stand-alone treatment and in addition to CPAP or mandibular devices being a non-invasive therapy with no side effects compared to other non-surgical techniques [4, 18–20]. Cranio-cervical extension may promote upper airway opening by elevating the hyoid bone, but it usually involves anteriorization of the head, which alters cervical posture. This compensation causes posterior traction of the hyoid bone, modifies the mandibular position, and reduces upper airway space, in addition to generating a dysfunctional pattern of cervical muscle activation [21]. The cervical spine exercises may contribute towards managing this disorder by improving the stability of related structures [22].

The objective of this study was to evaluate the efficacy of a program designed to combine TMO and cervical spine exercises in patients with mild to moderate OSA, analysing its impact on respiratory variables, daytime sleepiness, and quality of life [23].

## Materials and methods

### Study design

A stratified, longitudinal, prospective, single-blind, parallel-group, superiority clinical trial was conducted in the Department of Pneumology Sleep Unit at the ‘Marqués de Valdecilla’ University Hospital (HUMV) over a period of 19 months (May 2023–November 2024). Randomisation was stratified to guarantee a homogeneous gender-based distribution between the groups.

The sample size was calculated considering a clinically relevant minimal reduction of 30% of the Apnoea-Hypopnoea Index (AHI), using the study by Baz H et al. [24] which had the greatest sample size in the review by Camacho M [25]., with a 95% confidence level and 80% statistical power. This calculation estimated the need for 24 participants, assuming a 20% dropout rate.

The study was conducted in accordance with the human experimentation principles set out in the Declaration of Helsinki.

All operational procedures, implementation guidelines for the interventions and informed consent processes were approved by the Cantabria Ethics Committee for Research with Medicines and Medical Devices (CEIm), Santander, Spain. The registered protocol is available at http://clinicaltrials.gov/ (Identifier: NCT06681974).

### Selection criteria

The inclusion criteria were:

1. Diagnosis of OSA by polygraphy with mild to moderate AHI (5–30) without treatment criteria with CPAP and/or other treatments, such as mandibular advancement devices (MAD). Criteria defined by the Spanish consensus document [4].
2. Patients who refused CPAP or MAD.
3. Patients who were not candidates for CPAP or MAD for medical or technical reasons.
4. Age *≥* 18 years and *≤* 70 years.

The exclusion criteria were:

1. Craniofacial malformations.
2. Severe developmental delay and/or intellectual disability.
3. Diagnosis of neurodegenerative disease.
4. Limited tongue mobility or presence of ankyloglossia.
5. Temporomandibular joint disorder.
6. Regular use of hypnotic medications.
7. Bulbar pathologies.
8. Apnoea-hypopnoea index (AHI) of central origin for more than 50% of total sleep time.
9. Obesity grade II or higher.
10. Severe cardiovascular, neuromuscular or pulmonary pathology, or use of long-term home oxygen therapy. Obesity hypoventilation syndrome.

### Randomisation

Participants were randomly assigned using a password-protected Excel sheet with random codes (A1 or A2). “A1” corresponded to the experimental group and “A2” to the control group. The physiotherapist who trained the experimental group was the only person with access to the randomisation status. Intervention assignments for the study were blinded to the outcome assessors and study participants [26, 27]. 

### Intervention protocol

A polygraphic recording (apnoea-hypopnoea index, desaturation index, mean saturation and oxygen saturation time below 90%) was performed, and daytime sleepiness, quality of life and anthropometric variables of all participants (weight, height, body mass index and neck circumference) were measured.

Following this initial evaluation, they received an initial telephone call from the physiotherapist (Assessment 1-V1).

**Fig. 1:**
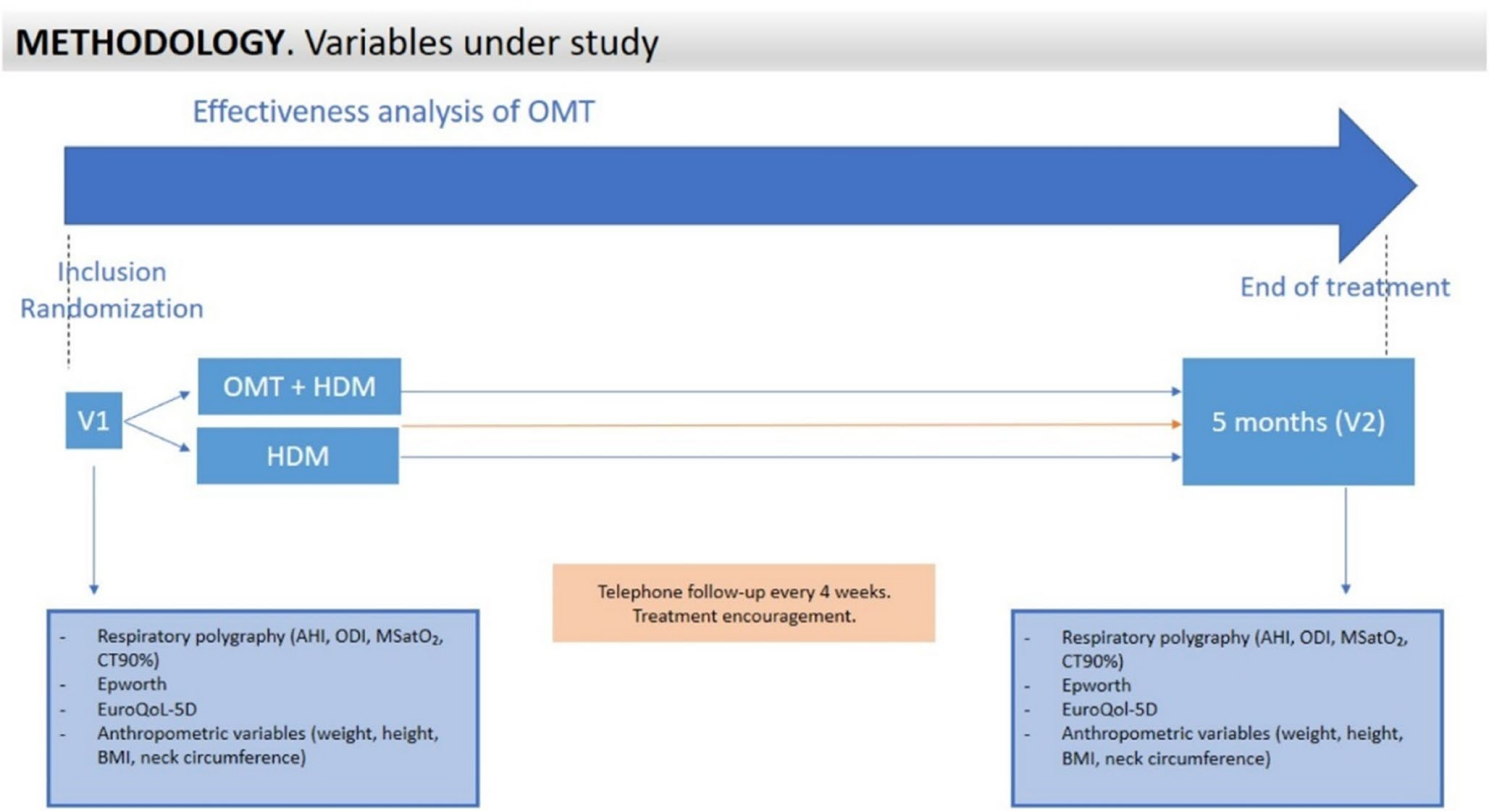
Flowchart of the study methodology

*Abbreviations: V1, Assessment 1; OMT, Oropharyngeal Myofunctional Therapy; HDM, Hygienic-Dietary Measures; treat., Treatment; BMI, Body Mass Index; AHI, Apnoea-Hypopnoea Index; ODI, Oxygen Desaturation Index; MSatO_2_, Mean Oxygen Saturation; CT90%, Time with Saturation Below 90%.

- Control group patients were reminded over the telephone of the Hygiene- and Diet-related Measures (HDM) consisting of establishing a regular sleep schedule, avoiding daytime naps, avoiding the use of screens or electronic devices, and adopting healthy lifestyle habits. These are detailed in Online Annex 1.

- In addition of being reminded of the HDMs, intervention group patients were asked to attend appointments to show them Oropharyngeal Myofunctional Therapy (OMT) consisting of exercises to strengthen the dilating muscles of the pharynx by way of tongue movements, the soft palate and the position of the hyoid bone, along with a set of cervical spine exercises. Cranio-cervical extension can facilitate upper airway opening by elevating the hyoid bone in patients with OSA, but it also alters cervical posture, reducing airway space and promoting dysfunctional muscle patterns, as described in the introduction. This group followed a structured OMT programme and cervical spine exercises over 20 weeks (3 sessions per week, 15–20 min per session). The intensity, duration and frequency of the exercises could be adjusted depending on each participant’s initial physical fitness. In all cases, patients had to meet the moderate intensity rating of perceived exertion (5–6 out of 10) according to the Modified Borg Scale [28]. The OMT and cervical spine exercise protocol, including the anatomical description of the levels involved (retropalatal, retroglossal, hypopharyngeal, and cervical spine), are described in Table 1 and detailed with images in the triptych in Online Annex 2. These participants received audiovisual support materials, and both the control and the intervention groups received telephone follow-up every four weeks. Once the therapy was complete, all subjects from the sample were re-evaluated (Assessment 2-V2). Figure 1 shows this procedure.

**Table 1:** Description of OMT exercise protocols

Level	Exercise/Phase	INITIAL Repetitions/Duration	Description
Retropalatal	Raise soft palate and uvula	1 set of 5 repetitions, hold for 3 to 4 s	Raise the soft palate and the uvula for 5 s
Retroglossal	Phase I: Slide tongue along the palate	1 set of 5 repetitions	From front to back along the palate
Retroglossal	Phase II: Suction with tongue against the palate	1 set of 5 repetitions, hold for 3 to 4 s	Strongly suction the tongue against the palate
Retroglossal	Phase III: Tongue against mouth floor	1 set of 5 repetitions, hold for 3 to 4 s	Press tip of the tongue against lower incisors
Retroglossal	Phase IV: Protrusion with tongue depressor	1 set of 5 repetitions, hold for 3 to 4 s	Press tongue against depressor
Hipopharyngeal	Inflate balloon with nasal breathing	2 set of 3 to 5 repetitions	Exhale through the mouth without removing the balloon
Hipopharyngeal	Chew and swallow	2 set of 3 to 5 repetitions	Keeping the tongue pressed against the palate
Cervical spine	Deep flexors (chin tuck)	2 set of 5 repetitions, hold for 3 to 4 s	Lying face upwards, draw a “C” with your earlobe. Craniocervical flexion.
Cervical spine	Deep extensors (head retraction)	2 set of 5 repetitions, hold for 3 to 4 s	Head straight backwards, “tuck chin into throat”

###  Primary Outcome Measures: Apnoea-Hypopnoea Index (AHI), Desaturation Index (DI), Mean Saturation (MSatO_2_), time with saturation below 90% (CT90%)

The initial assessment of sleep parameters was performed at the HUMV sleep laboratory using Type III Respiratory Polygraphy (RP) [29]. The total index of respiratory events was recorded, including the apnoea-hypopnoea index (AHI), the oxygen desaturation index (ODI), the mean oxygen saturation obtained throughout the recording, and the percentage of time with oxygen saturation below 90% (CT90%).

The available data were collected from the RPs performed on the sample subjects within the three months prior to inclusion in the study, and for those without prior RP, one was conducted before the start of the intervention. After completion of the programme, all sample subjects were reassessed through home RP, and the results were analysed by staff from the Sleep Unit (Fig. 1).

### Secondary outcome measures: daytime sleepiness and quality of life

An initial evaluation was made of both daytime sleepiness and quality of life using the Epworth Sleepiness Scale (ESS) and the EuroQol-5D Quality of Life Questionnaire (EQ-5D), respectively [30, 31]. The ESS was used to measure the level of daytime sleepiness of each patient. It consists of eight questions, each evaluating the likelihood of falling asleep in different everyday situations (score range from 0 to 24; 0–5 normal, 6–10 mild, 11–12 moderate, and 13–24 severe). The EQ-5D evaluates the patient’s quality of life through five dimensions and a visual analogue scale (VAS) related to health (scale from 0 = worst possible condition, to 100 = best possible condition). Both are detailed in Online Annex 3. These data were collected in person during the first visit (V1) by a member of the research team, who, once the participants completed the programme, also collected new data in person during the final visit (V2) (Fig. 1).

### Statistical analysis

The statistical analyses were performed using SPSS statistical software version 29.0 for Windows (IBM Corp). All the data were subject to descriptive statistics; continuous variables were reported as medians and interquartile ranges, and categorical variables as frequency counts and percentages. Levene’s test was used to determine the assumption of homoscedasticity. To evaluate both primary and secondary outcomes, the Mann-Whitney U test was used to compare hypotheses for independent non-parametric model samples in which the paired differences did not follow a normal distribution, and the significance level was set at *p* < 0.05.

## Results

### Recruitment and participant flow

A total of 65 participants were examined in the HUMV Sleep Unit between May 2023 and May 2024.

The main reasons for exclusion were obesity grade II or higher (*n* = 17; 68%), regular use of hypnotic medications (*n* = 6; 24%), and refusal to participate in the clinical trial despite meeting the inclusion criteria and not presenting any exclusion criteria (*n* = 2; 8%).

**Fig. 2:**
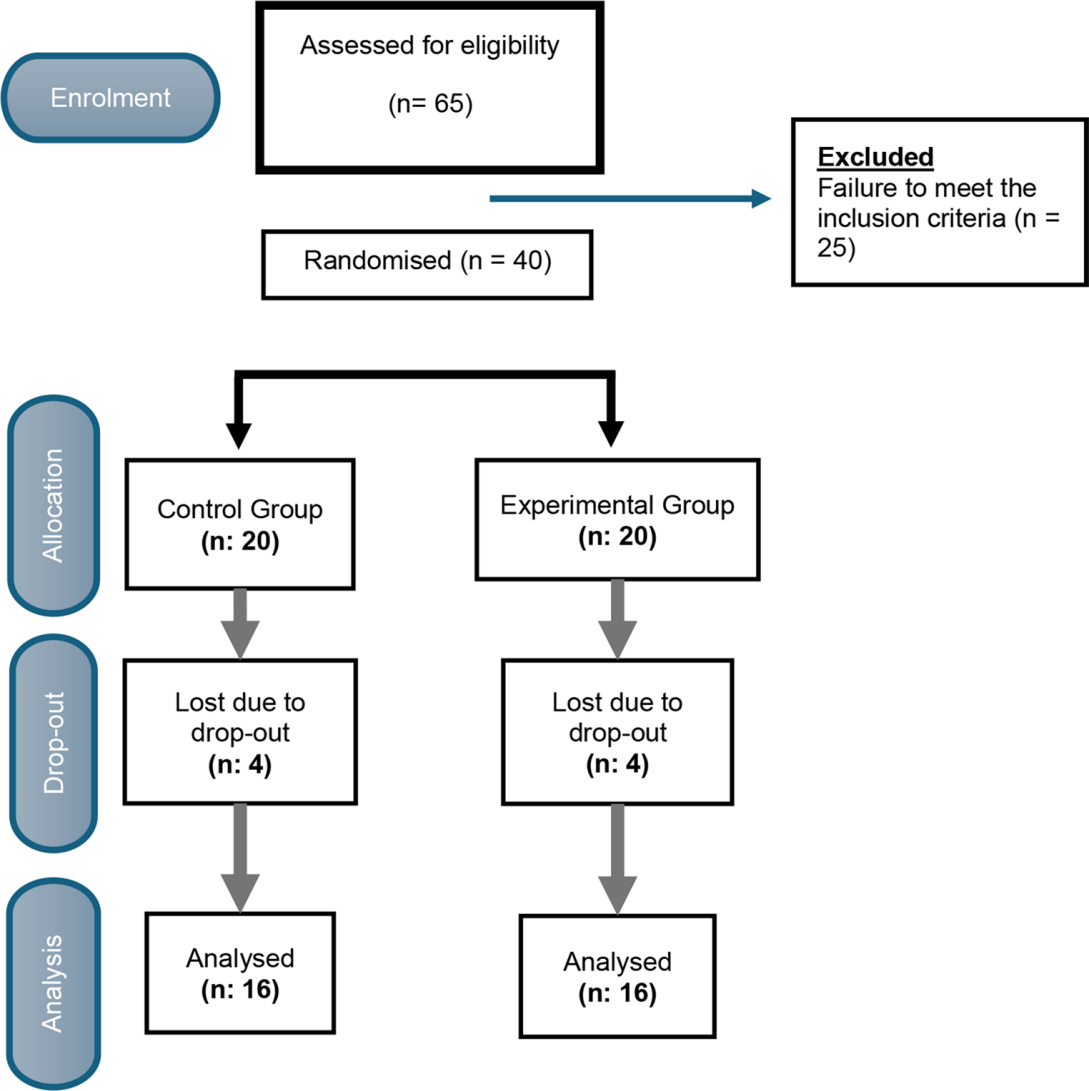
CONSORT flowchart of participants

Of the 40 participants who gave informed consent, *n* = 32 (80%) completed all evaluations. Figure 2 shows the participant flow throughout the study.

According to the experience of therapists who administered OMT, all participants who met the inclusion criteria (and none of those who met the exclusion criteria) demonstrated adequate coordination of oropharyngeal movement and sufficient muscle activation to perform the proposed exercises correctly.

Participants were divided into two groups:

- Group I Control: 20 adult patients with mild to moderate OSA.
- Group II Experimental: 20 adult patients with mild to moderate OSA.

### Baseline data

The initial demographic and clinical characteristics of the participants were comparable across all groups, as can be seen in Table 2. Distribution by gender, age and body mass index (BMI) of participants in the groups was not statistically significant (*p* > 0,05).

**Table 2:** Demographic characteristics of participants

		Total (*N* = 32)	Experimental (*N* = 16)	Control (*N* = 16)	*P*-value
Demographic Variables					
Gender, N *(%)*					
	Male	17 (*53.1*)	7 (*43.7*)	10 (*62.5*)	0.288
	Female	15 (*46.9)*	9 (*56.3*)	6 (*37.5*)	
BMI (Kg/m^2^), N *(%)*					
	Eutrophic (18.5–24.9)	8 (*25*)	5 (*31.2*)	3 (*18.8*)	0.771
	Pre-Obesity (25–29.9)	13 (*40.6*)	5 (*31.2*)	8 (*50*)	
	Obesity Class I (30–34.9)	11 (*34.4*)	6 (*37.6*)	5 (*31.2*)	
Age, N *(%)*					
	20–30	1 (*3.1*)	0 (*0*)	1 (*6.2*)	0.238
	31–40	6 (*18.7*)	2 (*12.5*)	4 (*25*)	
	41–50	11 (*34.4*)	8 (*50*)	3 (*18.8*)	
	51–69	14 (*43.8*)	6 (*37.5*)	8 (*50*)	
ANS, median *(IQR)*		38 (*36/40*)	38 (*36/40*)	38.5 (*37/41*)	0.616
Comorbidities, N *(%)*					
	HBP	6 (*18.7*)	1 (*6.2*)	5 (*31.2*)	0.074
	Dyslipidemias	5 (*15.6*)	2 (*12.5*)	3 (*18.7*)	0.643
	DM	0 (*0*)	0 (*0*)	0 (*0*)	
Scale and questionnaire					
EQ-5D, median (*IQR*)		70 (*59/74*)	70 (*52/77*)	70 *(59/74*)	0.956
ESS, median (*IQR*)		7 (*4/9*)	8.5 *(6/13*)	5 (*3/9*)	0.117

*Abbreviations: *BMI*, Body Mass Index; *ANS*, Average Neck Size; *HBP*, High Blood Pressure; *DM*, Diabetes Mellitus; *EQ-5D*, EuroQol-5D Quality of Life Questionnaire; *ESS*, Epworth Sleepiness Scale.

### Efficacy

**Fig. 3:**
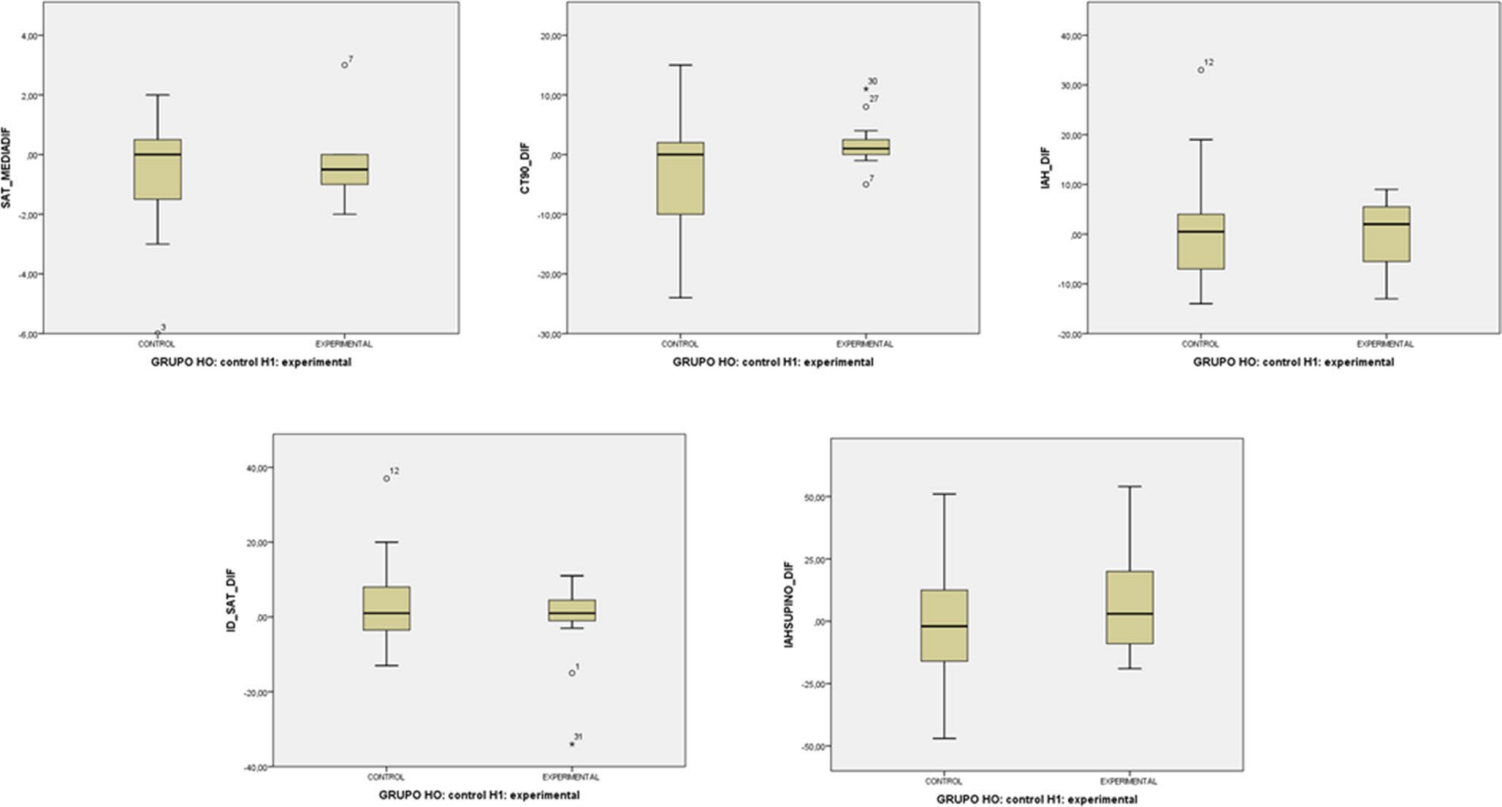
Box plot of primary outcome measures

**Fig. 4:**
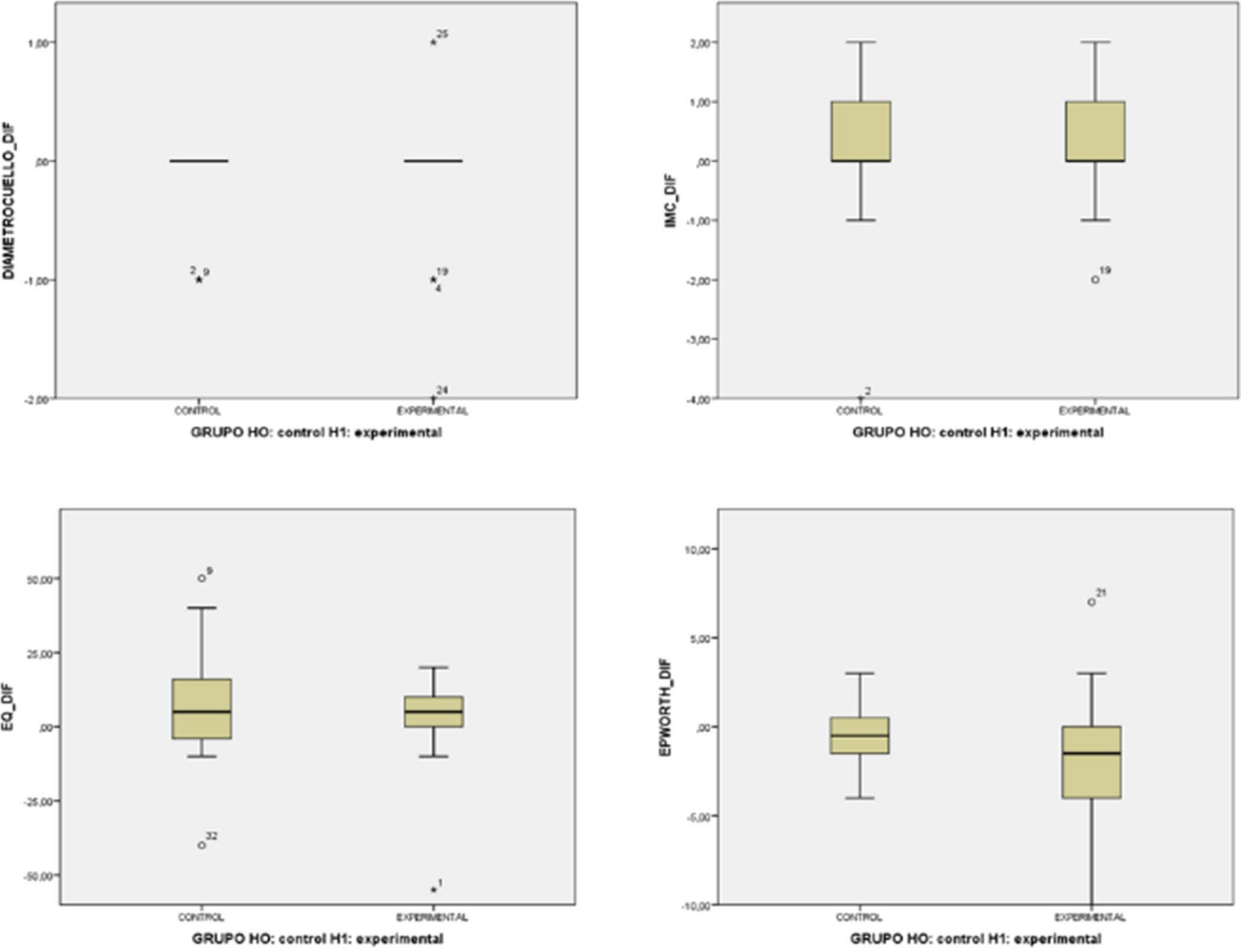
Box plot of secondary outcome measures

*Abbreviations: AHI, Apnoea-Hypopnoea Index; SatO_2_, Mean Oxygen Saturation; ODI, Oxygen Desaturation Index; TC90, Percentage of Time with Saturations below 90%; S AHI Supine Apnoea-Hypopnoea Index;

*Abbreviations: EQ-5D, EuroQol-5D Quality of Life Questionnaire; ESS, Epworth Sleepiness Scale, BMI, Body Mass Index; ANS, Average Neck Size.

After 20 weeks of treatment, no statistically significant changes were found in respiratory variables, sleepiness, quality of life, BMC or neck size. Only an insignificant tendency towards improved quality of life and sleepiness was observed in the experimental group with oropharyngeal exercise, as shown in Table 3 and in Figs. 3 and 4.

**Table 3:** Comparison of the different main variables of respiratory parameters and scales, median (IQR)

	Experimental G.				Control G.			*P*-value
	Pre-treat.	Post-treat.		Difference (Post-Pre)	Pre-treat.	Post-treat.	Difference (Post-Pre)	
AHI	13.5 (9/18)	14.5 (9/18)		2.0 (−6/6)	12.5 (11/21)	12.0 (9/18)	0.5 (−7/4.5)	0.86
MSatO_2_	93.0 (93/94)	93.0 (92/94)		−0.5 (−1/0)	93.0 (92/94)	93.0 (92/94)	0.0 (−2/1)	0.43
ODI	12.5 (7/18)	13.5 (19/20)		1.0 (−1/5)	11.5 (10/18)	11.5 (8/19)	1.0 (−5/9)	0.72
TC90	1.5 (0/3)	3.0 (0/7)		1.0 (0/3)	2.5 (0/17)	1.0 (0/6)	0.0 (−10/2)	0.10
S AHI	17.0 (14/29)	23.0 (10/41)		3.0 (−9/21)	21.0 (14/32)	19.0 (8/34)	−2.0 (−17/14)	0.38
EQ-5D	70.0 (52/77)	72.5 (52/87)		5.0 (0/10)	70.0 (59/74)	77.5 (62/80)	5.0 (−6/18)	0.08
ESS	8.5 (6/13)	7.0 (4/10)		−1.5 (−4/0)	5.0 (3/9)	5.0 (3/7)	−0.5 (−2/1)	0.83
BMI	28.5 (23/31)	28.0 (24/32)		0.0 (0/1)	28.0 (25/31)	27.5 (25/31)	0.0 (0/1)	0.93
ANS	38.0 (36/40)	37.5 (36/40)		0.0 (0/0)	38.5 (37/41)	38.5 (37/41)	0.0 (0/0)	0.79

*Abbreviations: *AHI*, Apnoea-Hypopnoea Index; *SatO2*, Mean Oxygen Saturation; *ODI*, Oxygen Desaturation Index; *TC90*, Percentage of Time with Saturations below 90%; *S AHI* Supine Apnoea-Hypopnoea Index; *EQ-5D*, EuroQol-5D Quality of Life Questionnaire; *ESS*, Epworth Sleepiness Scale, *BMI*, Body Mass Index; *ANS*, Average Neck Size.

*Pre- and post-treatment values are medians (range); differences are means of individual change.

## Discussion

This study evaluated the combination of oropharyngeal and cervical spine strengthening exercises (3 times a week for 20 weeks) in patients with OSA, adapted to their functional and clinical characteristics (OMT and cervical exercises represent low-risk therapeutic approaches, offering a viable and less invasive alternative for improving oropharyngeal function and posture). The 2022 international consensus document on OSA by the Spanish Society of Pneumology and Thoracic Surgery (SEPAR) recommends OMT in mild to moderate, non-obese OSA as support for CPAP and with MAD [4]. The results did not show a significant reduction in the severity of OSA or relevant improvements in respiratory parameters; furthermore, no favorable trend was observed in the experimental group compared to the control group. On the contrary, in several of these indicators, the evolution was slightly more favorable in the control group, although without reaching statistical significance. However, clinical relevance was identified in two key variables: on the one hand, an improvement in daytime sleepiness in the experimental group; and on the other, an improvement in quality of life in both groups, possibly associated with the implementation of sleep hygiene measures. These findings suggest clinically positive effects that deserve to be taken into account, even in the absence of statistically significant differences. Defining the optimal treatment remains a challenge, and although the exercises were well tolerated, they failed to show significant benefits at the frequency, duration and intensity of the and protocol studied. This research focused exclusively on strengthening exercises targeting the oropharyngeal and cervical muscles, considering the balanced composition of muscle fibers in the dilator muscles of the pharynx, which are composed of approximately 50% type I fibers (slow twitch) and 50% type II fibers (fast twitch) [32, 33].

The incorporation of cervical exercises, as discussed in the introduction, is justified by biomechanical principles. Cranio-cervical extension can promote the opening of the upper airways by elevating the hyoid bone; however, this maneuver often involves anteriorization of the head, which alters cervical postural alignment. This postural compensation induces posterior traction of the hyoid bone, modifies the position of the mandible, and reduces the space available in the upper airways, as well as promoting a dysfunctional pattern of activation in the cervical muscles. In this regard, it may be more effective to preserve the physiological cervical curvature by strengthening the deep cervical flexor and extensor muscles, as well as the suprahyoid muscles (to facilitate a higher position of the hyoid) and the pharyngeal dilator muscles, rather than resorting to craniocervical extension as a compensatory strategy [21, 34].

The results of the training protocol conducted during this study do not match the recognised benefits of OMT in reducing OSA severity, as some reviews report reductions in AHI of up to 50% in adults receiving OMT [25]. However, more recent reviews such as the one by Rueda et al. [12], indicate that OMT could improve daytime sleepiness and the short-term sleep quality compared to standard medical treatment, but may not offer significant differences compared to CPAP and could even increase AHI. The available evidence is insufficient and its effectiveness limited to recommend it as a standard treatment—findings that align with our study.

Adherence to the treatment protocol based on the OMT concept was an area of particular interest. Most of the articles published do not provide data on adherence to treatment in the experimental group [35, 36], and some of the ones that do even refer to adherence as low as 50% [37, 38]. Some of the factors favouring adherence to exercise, such as their short-term duration, the frequency with which they are performed and audiovisual support, were included in this study to create a more attractive protocol, seeking to generate the minimum drop-out rate in the experimental group, as these exercises were to be performed at home. However, the absence of objective and measurable adherence monitoring (such as digital records, electronic devices, or direct supervision by therapists) limits the ability to differentiate between treatment ineffectiveness and low fidelity to the intervention. Since it is not possible to verify compliance by participants, it cannot be ensured that negative results truly reflect the ineffectiveness of the protocol. Although the use of objective adherence monitoring tools could improve the interpretability of the results and provide a more accurate view of the treatment’s effectiveness, their implementation in studies such as this one would require considerable material and human resources.

The frequency of OMT execution tends to be very high—reanging from 1 to 3 times per day, and up to 4 to 7 days per week, simulating or reproducing the original conditions of the early exercise protocols proposed by Guimarães et al. [39]. This study reduced the frequency of OMT to ensure individualised completion and avoid drop-outs while maintaining adequate intensity. Although other studies replicated prior protocols with high adherence, correct application of the exercises was prioritised here, providing tools to optimise their effectiveness on the UA muscles.

Prior studies on OMT lasted between 8 and 16 weeks, with an average of 12 weeks and demonstrated benefits in OSA [39–41]. Our 20-week study expanded the follow-up for a better analysis of its impact, aligning with reviews that suggest longer studies [12, 25]. However, the key difference seems to be the high frequency of performance, as shorter studies did report improvements in OSA severity. The duration of the exercises (15–20 min) was similar to that in other research (8–30 min). Although it was unknown whether this approach would reduce OSA, its study was relevant.

Therefore, the lack of changes in AHI and other variables could be due to the low frequency of OMT, as a high frequency seems key in reducing OSA severity and sleepiness and in improving quality of life. In a therapeutic or rehabilitation context, exercising 1 to 3 times per week is considered low frequency, while exercising 5 or more times per week, or even several times a day, is considered high frequency. Individually adjusting intensity may not be sufficient for a measurable effect. A similar study by Poncin et al. [36] (OMT 4 days per week for 6 weeks) also failed to show an impact on OSA, although it did reduce daytime sleepiness. The main difference with this study is its duration (20 weeks), suggesting that frequency is a crucial factor.

The main strength of this study is its long-term follow-up, which was lacking in prior trials. Furthermore, the inclusion/exclusion criteria were appropriately defined to control for bias, stratified blinded randomisation was used, and the sample size was increased to reduce random errors and unknown factors. However, there are limitations: this is a single-centre study, which reduces generalisation, and objective means of measuring adherence were not used, unlike other studies with electronic records [36, 42]. Furthermore, participant loss and time restrictions may have affected the study’s validity.

The results of this study underline the complexity inherent to the design and implementation of non-invasive interventions for the treatment of OSA. Despite incorporating solid methodological elements (such as extended follow-up, strict inclusion criteria and a protocol tailored to the individual characteristics of patients), the absence of significant effects on major clinical parameters suggests that the frequency of OMT performance may play a determining role in its efficacy.

These findings confirm that treatment duration alone is not sufficient without adequate intensity and frequency—elements that seem to be closely linked to therapeutic effectiveness in this population. To this end, this study provides relevant evidence not only of the limitations of certain intervention strategies but also of the need to establish standardised criteria to optimise the clinical application of OMT. In the future, it will be vital to develop multicentre studies using comparative methodologies and long-term follow-up, which systematically evaluate the interaction between frequency, duration, adherence and clinical outcomes. Only through a deeper understanding of these factors will it be possible to advance towards personalised, effective and sustainable treatments for patients with OSA.

## Conclusions

Our results indicate that a 20-week program of OMT and cervical exercises did not produce significant differences in the severity of OSA compared to lifestyle and dietary measures. In addition, some respiratory parameters evolved more positively in the control group, although without reaching statistical significance. Clinical relevance was only observed in the reduction of daytime sleepiness in the experimental group and in the improvement of quality of life in both groups, possibly related to sleep hygiene. These results do not support the efficacy of the protocol applied under the conditions evaluated and suggest the need to review its approach before considering future research.

## Supplementary Information

Below is the link to the electronic supplementary material.

Supplementary Material 1 (DOCX. 1.04 MB)

Supplementary Material 2 (DOCX. 120 KB)

Supplementary Material 3 (DOCX. 16.0 KB)

## Data Availability

The datasets generated and/or analysed during the current study are securely stored in the hospital’s repository and are available for collaborative and research purposes by contacting the corresponding author.
