# Supplementary material for: Effectiveness of strenghtning oropharyngeal myofunctional therapy combined with cervical spine exercises in mild to moderate obstructive sleep apnoea
Source: Sleep Breath. 2025 Nov 8;29(6):348. doi: 10.1007/s11325-025-03487-w (PMC12596285; doi:10.1007/s11325-025-03487-w)
Supplement: Supplementary file 1 — Supplementary Material 1 (DOCX. 1.04 MB) [file 11325_2025_3487_MOESM1_ESM.docx]

**OROPHARYNGEAL EXERCISES**

**RETROPALATAL LEVEL**

1 set of 5 repetitions.


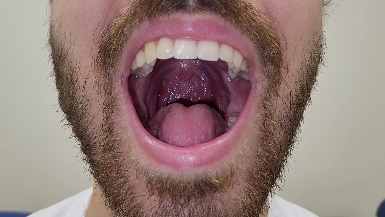
Raise the soft palate and uvula for 5 seconds.

**RETROGLOSSAL LEVEL**

1 set of 5 repetitions.

**Phase I**


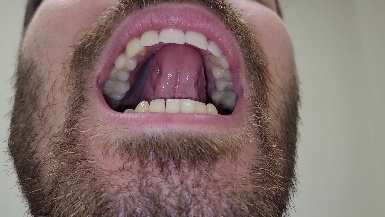
Slide the tip of your tongue over your palate from front to back.

**Phase II**


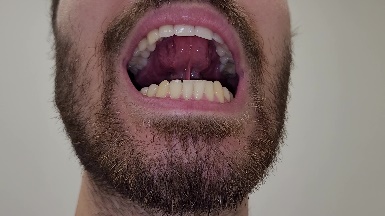
Strongly suction while keeping your tongue pressed against your palate.

**Phase III**


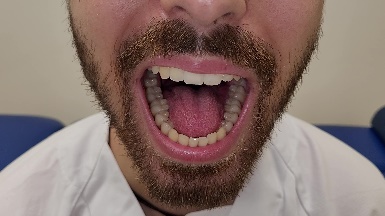
Press your tongue against the floor of your mouth, holding the tip against your lower incisors.

**Phase IV**

**
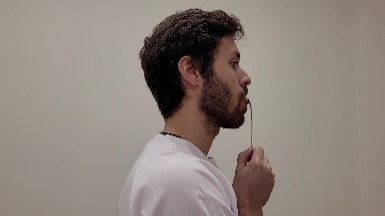
**Protrude your tongue against a tongue depressor for 3 to 4 seconds).

**HYPOPHARYNGEAL LEVEL**

**Breathing**

3 to 5 repetitions.


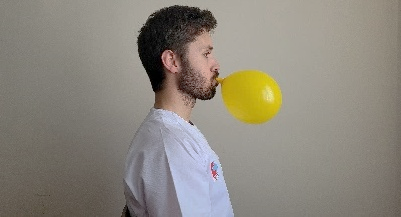
Inflate a balloon by nasal inhalation and release the air through your mouth, without removing the balloon from your mouth.

**Chewing and swallowing**

3 to 5 repetitions.


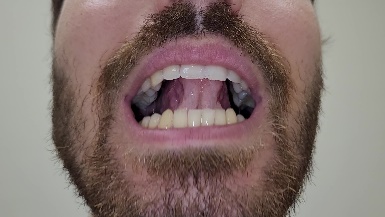
Alternate chewing and swallowing while keeping your tongue against your palate.

**CERVICAL SPINE LEVEL**

**Deep flexor muscles**

5 repetitions of 2 to 3 seconds.


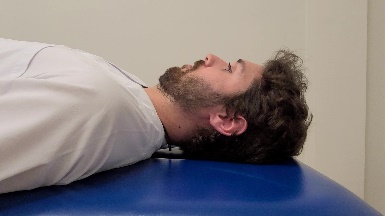
Chin tuck: lying face upwards, slowly “draw a C” with your earlobe.

**Deep extensor muscles**

5 repetitions.


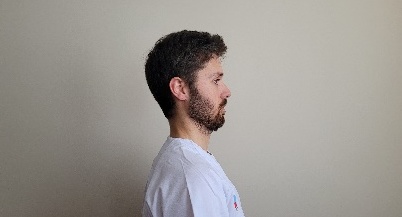
Retraction movement in which the head moves straight backwards, “tucking your chin into your throat”.
