# Supplementary material for: Effectiveness of strenghtning oropharyngeal myofunctional therapy combined with cervical spine exercises in mild to moderate obstructive sleep apnoea
Source: Sleep Breath. 2025 Nov 8;29(6):348. doi: 10.1007/s11325-025-03487-w (PMC12596285; doi:10.1007/s11325-025-03487-w)

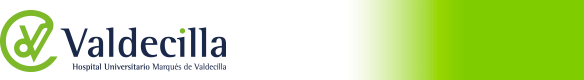


**Sleep and Ventilation Disorders Unit**

SLEEP HYGIENE GUIDELINES

-
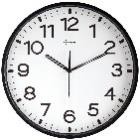
**Establish a regular sleep schedule.** It is important to wake up and go to bed at the same time every day, even if you haven’t slept well the night before. Avoid altering this schedule on weekends or during holidays.
-
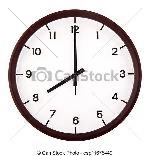
Don’t stay in bed longer than your usual schedule allows, even if you woke up several times during the night or slept poorly. The sleep-wake cycle regulates our sleep needs, and staying in bed longer may negatively affect sleep the following night
-
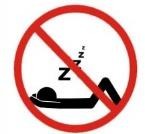
Avoid daytime naps. If you must nap, limit it to 20–30 minutes. Use an alarm or ask someone to wake you — never rely on waking up naturally.
-
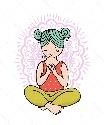
**Avoid stimulating or physically/mentally demanding activities** in the three hours before bedtime (such as sports, dancing, physical exercise, studying, or intense reading). Schedule these activities for earlier in the day, preferably in the morning or early afternoon. Instead, develop relaxing bedtime habits, like reading a simple book in a quiet, dimly lit environment. Engaging in demanding tasks at night will interfere with your ability to fall asleep.
- **Avoid screen use (phones, tablets, computers, TVs)** and exposure to bright light in the three hours before bedtime. This interferes with melatonin production — a hormone that regulates the sleep-wake cycle and is influenced by light exposure. Being exposed to light when the body expects darkness disrupts its synthesis.


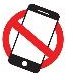

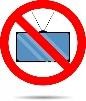


- **Follow a healthy bedtime routine.** Eat dinner 2 to 3 hours before going to sleep. Dinner should be light but filling. Avoid going to bed hungry or thirsty. Refrain from consuming stimulants (coffee, chocolate, tea, etc.) late in the afternoon or within three hours of bedtime. Taking a warm bath or shower may help you relax before bed
-
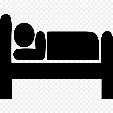
**Associate the bedroom and bed exclusively with sleep.** Avoid starting sleep elsewhere. Keep TVs, computers, or tablets out of the bedroom. Don’t eat, watch TV, or work in bed. Maintain a quiet, comfortable sleeping environment with an appropriate temperature (not too hot or cold) and complete darkness (avoid keeping lights on while sleeping).
-
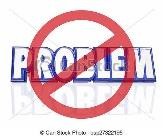
**Avoid “taking your problems to bed.”** When going to bed, try to disconnect from worries. Plan to address them the next day, not while trying to fall asleep. Don’t focus on your inability to sleep or check the clock. Instead, focus on progressively relaxing your body and mind.
-
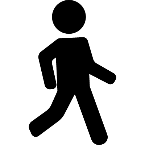
**If you wake up during the night, don’t stay in bed.** Try not to get anxious. Leave the bed and go to a quiet space. Do something relaxing and ideally boring in dim lighting (e.g., peaceful reading in the living room). Avoid eating or doing anything physically or mentally stimulating. Return to bed only when you feel sleepy again
- **Maintain healthy lifestyle habits.** Engage in at least one hour of regular physical activity daily, preferably outdoors in sunlight (but not within three hours of bedtime). Physical activity and light exposure promote melatonin production and help regulate the sleep-wake cycle. Stay physically and mentally active throughout the day to reduce daytime drowsiness. Avoid tobacco, alcohol, and other harmful substances. Refrain from consuming chocolate, coffee, tea, and energy drinks late in the day or before bedtime


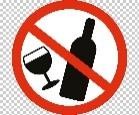

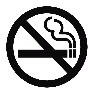

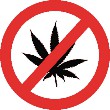

Supplement: Supplementary file 2 — Supplementary Material 2 (DOCX. 120 KB) [file 11325_2025_3487_MOESM2_ESM.docx]
