# Supplementary figures and images for: Effectiveness of strenghtning oropharyngeal myofunctional therapy combined with cervical spine exercises in mild to moderate obstructive sleep apnoea
Source: Sleep Breath. 2025 Nov 8;29(6):348. doi: 10.1007/s11325-025-03487-w (PMC12596285; doi:10.1007/s11325-025-03487-w)

SOMNOLENCIA: Epworth


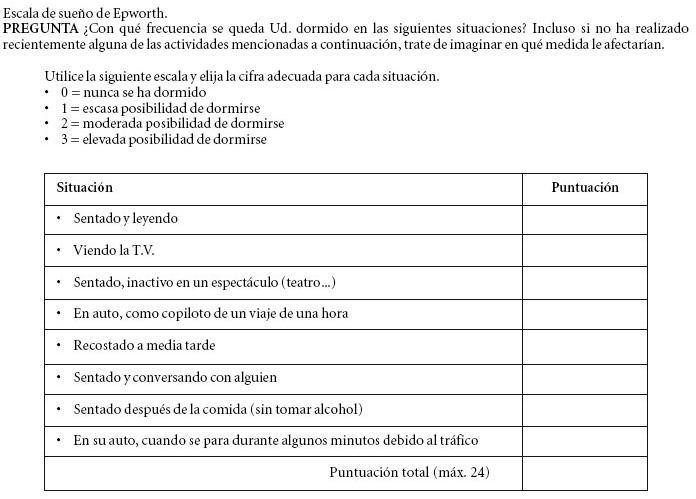


CALIDAD DE VIDA: EuroQol 5D ([www.](http://www/) euroqol.org)


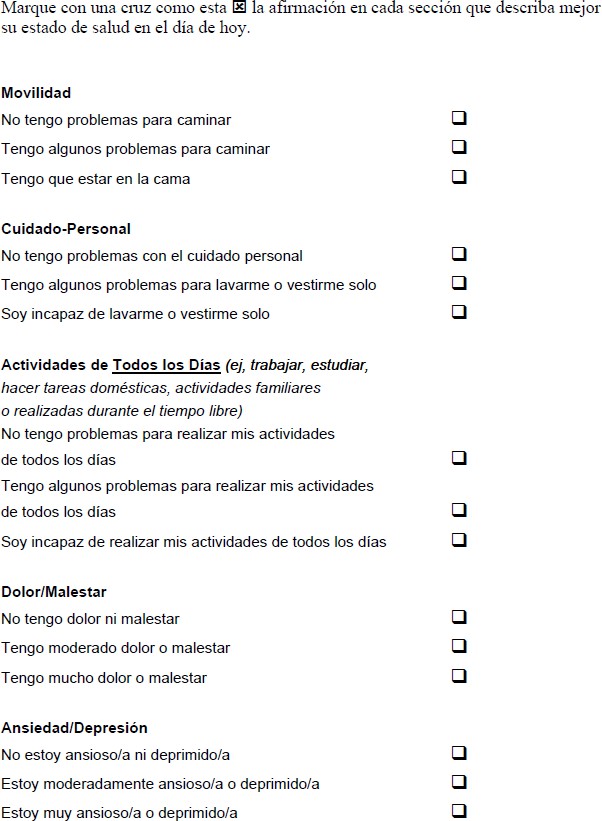


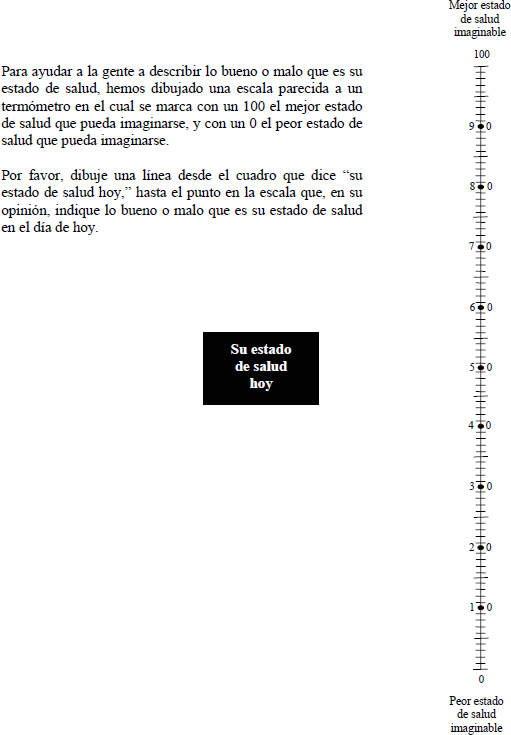

Supplement: Supplementary file 3 — Supplementary Material 3 (DOCX. 16.0 KB) [file 11325_2025_3487_MOESM3_ESM.docx]
